# Synthesis and microwave absorption properties of electromagnetic functionalized Fe_3_O_4_–polyaniline hollow sphere nanocomposites produced by electrostatic self-assembly

**DOI:** 10.1007/s11051-013-1988-4

**Published:** 2013-09-24

**Authors:** Yao-Feng Zhu, Qing-Qing Ni, Ya-Qin Fu, Toshiaki Natsuki

**Affiliations:** 1 Key Laboratory of Advanced Textile Materials and Manufacturing Technology Ministry of Education, Zhejiang Sci-Tech University, Hangzhou, 310018 Zhejiang People’s Republic of China; 2 Department of Functional Machinery and Mechanics, Shinshu University, Tokida, Ueda, 386-8576 Japan

**Keywords:** Fe_3_O_4_ nanoparticles, Polyaniline hollow sphere, Nanocomposite, Polyelectrolyte, Microwave absorption

## Abstract

Highly regulated Fe_3_O_4_–polyelectrolyte-modified polyaniline (Fe_3_O_4_–PE@PANI) hollow sphere nanocomposites were successfully synthesized using an electrostatic self-assembly approach. The morphology and structure of the Fe_3_O_4_–PE@PANI nanocomposites were characterized using field-emission scanning electron microscopy, transmission electron microscopy, Fourier-transform infrared spectroscopy, X-ray powder diffraction, thermogravimetric analysis, and X-ray photoelectron spectroscopy. The results showed that the as-prepared nanocomposites had well-defined sizes and shapes, and the average size is about 500 nm. The assembly process was investigated. Magnetization measurements showed that the saturation magnetization of the nanocomposites was 38.6 emu g^−1^. It was also found that the Fe_3_O_4_–PE@PANI nanocomposites exhibited excellent reflection loss abilities and wide response bandwidths compared with those of PANI hollow spheres in the range 0.5–15 GHz. The Fe_3_O_4_–PE@PANI nanocomposites are, therefore, promising for microwave absorption applications.

## Introduction

Combinations of conducting polymers and inorganic magnetic nanoparticles have recently attracted significant interest because the resultant materials exhibit both conductive and magnetic properties, and take advantage of the properties of both conducting polymers and inorganic nanoparticles. Electromagnetic functionalized conducting polymer nanocomposites have great potential for applications in microwave-absorbing materials, electrochemical displays, nonlinear optics, and electromagnetic shielding (Shen et al. 2010; Zhou et al. 2011; Kang et al. 1998; Kawaguchi 2000; Gomez-Romero 2001; Zhang and Wan 2003; Marchessault et al. 1992; Fang et al. 2011). Interest in the design and controlled fabrication of materials with specific conducting and magnetic properties, therefore, continues to grow. Polyaniline (PANI), which is an excellent conducting polymer, has been known for more than a century and studied in many fields because of its excellent environmental stability and ease of doping (MacDiarmid 2001). Particularly, PANI-based nanocomposite, the most important material for the twenty-first century, has received special attention owing to their potential wide applications arising from the unique nanofiller-introduced thermal stability, electrochemical, mechanical, magnetic, and dielectric properties (Zhang et al. 2013). For example, grapheme/PANI (Wei et al. 2012a, b), BaTiO_3_/PANI (Zhang et al. 2013; Zhu et al. 2013), and multi-walled carbon nanotube/PANI (Wei et al. 2013; Gu et al. 2013) for supercapacitors, stealth materials, and environmental remediations have been recently explored and investigated (Wei et al. 2012a, b).

As magnetic nanoparticles, magnetite (Fe_3_O_4_) nanoparticles are mostly investigated among the many magnetic materials owing to their interesting magnetic properties and are easy to synthesize, and have a wide range of potential applications in various fields, such as magnetic recording media, photonic crystals, microwave-absorbing materials, and biomedical applications (Wei et al. 2012a, b; Umare et al. 2010; Zhang et al. 2011; Kim et al. 2008). Recently, various Fe_3_O_4_–PANI micro/nanostructures have been the focus of research because their properties are different from those of the corresponding bulk forms. Shape-controlled synthesis of Fe_3_O_4_–PANI nanocomposites with desired morphologies is, therefore, a hot research topic. Conducting polymer hollow spheres have potential applications in reactors, catalysts, sensors, carriers, combinatorial analytics, and photochemistry (Meier 2000; Shchukin and Sukhorukov 2004; Peyratout and Dahne 2004), and are also promising as ideal microwave resonators because of their special structures, low densities, and light weights. The development of microwave absorbers has been an important technology for eliminating electromagnetic wave pollution. Recently, the demand for various microwave absorbers for commercial and military applications has increased. The preparation of Fe_3_O_4_–PANI hollow sphere nanocomposites for use as microwave absorbers is, therefore, of interest.

In the past few decades, various techniques have been developed for the fabrication of PANI and magnetic nanocomposites, mainly by in situ synthesis of a conjugated polymer via oxidative (Deng et al. 2003) and electrochemical oxidation polymerizations (Bidan et al. 1994). However, most electromagnetic functionalized nanocomposites prepared by these processes typically produce an uncontrolled structure and unpredictable material properties. It is, therefore, important to find methods of fabricating Fe_3_O_4_–PANI nanocomposites with desired shapes and high Fe_3_O_4_ contents. Electrostatic self-assembly has been shown to be a promising approach to produce large-scale periodic structures with the desired properties. This method permits the fabrication of thin-film assemblies on solid supports by spontaneous sequential adsorption of oppositely charged species from dilute aqueous solutions onto charged substrates (Caruso et al. 1998; Liu and Choi 2012).

In the present work, we describe a facile, general, eco-friendly, and effective approach to the fabrication of Fe_3_O_4_–PANI hollow sphere nanocomposites based on polyelectrolyte-modified PANI hollow spheres (PE@PANI). We then explore the potential application of the as-prepared Fe_3_O_4_–polyelectrolyte-modified PANI hollow sphere (Fe_3_O_4_–PE@PANI) nanocomposites as microwave absorbers. The morphology, structure, magnetic properties, and formation mechanism of the Fe_3_O_4_–PE@PANI nanocomposites were investigated in detail. To the best of our knowledge, this is the first report of Fe_3_O_4_–PE@PANI hollow sphere nanocomposites prepared by electrostatic self-assembly.

## Experimental section

### Materials

Analytical grade aniline (99 %), H_2_O_2_ (30 %), FeCl_3_·6H_2_O, FeCl_2_·4H_2_O, ammonia (28 %), and ethanol (99.5 %) were purchased from Wako Pure Chemical Industries, Ltd., Osaka, Japan. Analytical grade poly(sodium 4-styrenesulfonate) (PSS, average *M*_w_ ~ 70,000) and poly(allylamine hydrochloride) (PAH, average *M*_w_ ~ 56,000) were purchased from Sigma-Aldrich, St Louis, MO, USA. Deionized water was used in all the experiments. All chemicals were used without further purification.

### Synthesis of Fe_3_O_4_ nanoparticles

The Fe_3_O_4_ magnetic nanoparticles were prepared using a modified co-precipitation method. The reaction was carried out in a 250-mL three-necked round-bottomed flask equipped with a stirrer. FeCl_2_·4H_2_O (0.994 g, 5 mmoL) in 10 mL of deionized water and FeCl_3_·6H_2_O (2.73 g, 10 mmoL) in 10 mL of deionized water were mixed by vigorous stirring, and the mixed solution was kept in a water bath at 80 °C. Preheated ammonia solution (1.5 M, 20 mL) was added rapidly to the solution, followed by dropwise addition of aqueous ammonia, with stirring, until the pH reached 10–12; stirring was then continued for 1 h. The Fe_3_O_4_ nanoparticles formed were collected by magnetic field separation, washed three times with deionized water, and dried under vacuum at 50 °C for 24 h.

### Synthesis of Fe_3_O_4_–PE@PANI hollow sphere nanocomposites

PANI hollow spheres were synthesized according to the method reported in the literature (Zhang et al. 2009a, b): 1 mmoL (0.1 mL) of aniline monomer was added to 40 mL of aqueous H_3_PO_4_ solution (0.4 M) at room temperature, with vigorous stirring, over several minutes, to form a uniform solution. Then 0.12 mL of H_2_O_2_ and 0.02 mL of FeCl_3_ (0.1 M) aqueous solution, in turn, were mixed with this solution. After fully mixing by stirring, the mixture was transferred to a Teflon-lined stainless-steel autoclave. The autoclave was sealed and maintained at 140 °C for a specific time. After immediately cooling to room temperature, the precipitate was filtered and washed several times with deionized water and ethanol, and dried under vacuum at 60 °C for 12 h.

The as-prepared PANI hollow spheres were immersed in 0.5 M NaCl aqueous solution (pH = 4) with ultrasonication for 20 min. PAH was then added to the mixture to give a final concentration of 1 mg mL^−1^. The PAH adsorption time was 20 min under ultrasonication. Excess PAH was removed by three centrifugation/washing/redispersion cycles. Negatively changed PSS was then deposited on the coated PANI hollow spheres using the same conditions and procedures. Finally, the polyelectrolyte-coated PANI hollow spheres (PE@PANI) were formed.

The Fe_3_O_4_–polyelectrolyte-modified PANI hollow sphere (Fe_3_O_4_–PE@PANI) nanocomposites were formed by adding 1 mg mL^−1^ of an Fe_3_O_4_ uniformly magnetic fluid to PE@PANI dispersed in deionized water under the adsorption conditions (pH = 4), allowing 20 min for Fe_3_O_4_ adsorption, and removing excess Fe_3_O_4_ by three centrifugation/washing/redispersion cycles. The precipitate was dried under vacuum at 60 °C for 24 h.

### Characterization

The zeta (ζ)-potentials of the different samples were measured using a Zetaplus analyzer (Nano ZS, Malvern Instruments Ltd., Malvern, UK) by an electrophoretic light-scattering method. The morphologies and microstructures of the products were characterized using field-emission scanning electron microscopy (FE-SEM; JEOL S-5000, JEOL Ltd., Tokyo, Japan) and transmission electron microscopy (TEM; JEOL JEM-2010) with an accelerating voltage of 200 kV. The crystal structures of the prepared powders were analyzed by X-ray diffraction (XRD; RINT 2550H diffractometer) using Cu Kα radiation. Fourier-transform infrared (FT-IR) spectra were obtained with a Shimadzu IR Prestige-21 spectrometer (Shimadzu, Kyoto, Japan) using KBr pellets. Thermogravimetric analysis (TGA; TG8120, Rigaku Denki, Tokyo, Japan) was carried out under an air atmosphere from room temperature to 600 °C at a heating rate of 5 °C min^−1^. X-ray photoelectron spectroscopy (XPS; Kratos AXIS Ultra DLD) was used to analyze the chemical compositions and chemical states of the samples. Magnetic measurements were carried out at room temperature using a vibrating sample magnetometer (TM-VSM5250, Japan) with a maximum magnetic field of 10 kOe.

**Scheme 1:**
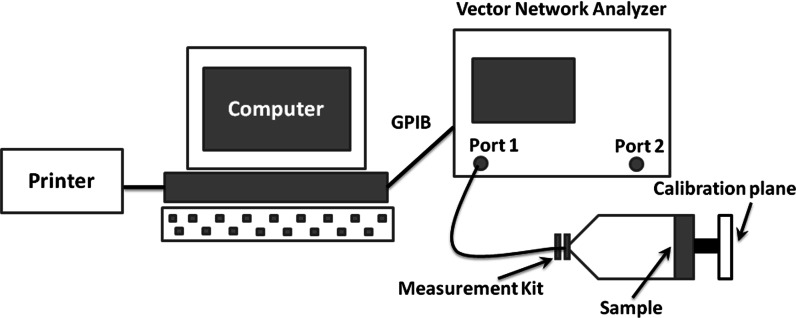
Schematic of test system

The real and imaginary parts of the complex permittivity *ε* (*ε* = *ε′* − *jε*″) and permeability *μ* (*μ* = *μ*′ − *jμ*″) were measured using a vector network analyzer (37247D, Anritsu Co., Ltd.) over the range 0.5–15 GHz. The test system is shown in Scheme 1. The samples consisted of Fe_3_O_4_–PE@PANI nanocomposites loaded in paraffin with different weight fractions. The powder–wax compound was pressed into a toroidal shape with an outer diameter of 7 mm, inner diameter of 3 mm, and a thickness of 2 mm; the reflection loss was calculated from the measured complex permittivity and permeability.

## Results and discussion

### Design of synthesis strategy

**Fig. 1:**
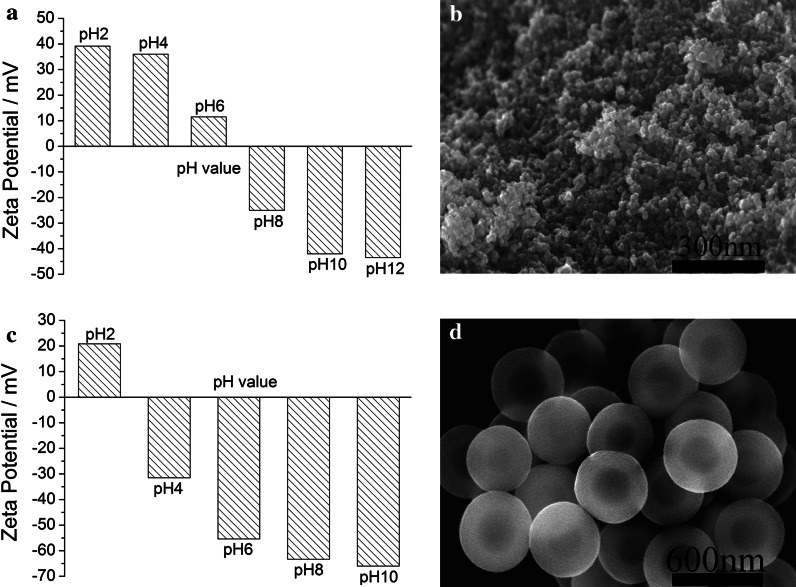
Values of ζ-potential at different pH values for **a** Fe_3_O_4_ nanoparticle aqueous solution and **c** PANI hollow sphere aqueous solution. FE-SEM images of **b** Fe_3_O_4_ nanoparticles and **d** PANI hollow spheres

**Scheme 2:**
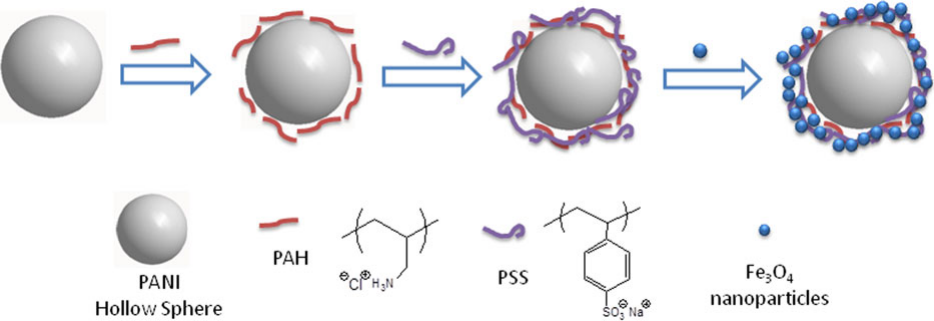
Schematic illustration of synthesis of Fe_3_O_4_–PE@PANI nanocomposites

Our strategy for the synthesis of Fe_3_O_4_–PE@PANI nanocomposites consisted of two main steps: (1) grafting of polyelectrolyte chains onto PANI hollow sphere surfaces to prepare nanocatchers and (2) assembling Fe_3_O_4_ nanoparticles on the polyelectrolyte-coated PANI hollow spheres (the so-called nanocatchers). Figure 1 shows the ζ-potentials at different pH values in aqueous solution and the morphologies of the Fe_3_O_4_ nanoparticles and PANI hollow spheres. As shown in Fig. 1a and b, the isoelectric point of the Fe_3_O_4_ nanoparticles is about 6.6, and the average size is about 10–20 nm. Figure 1c and d indicates that the isoelectric point of the PANI hollow spheres is about 2.8, and the average size is about 450 nm. These results provide guidance with regard to the conditions for the synthesis of the Fe_3_O_4_–PE@PANI nanocomposites. Evidently, the best conditions for adsorption are an aqueous solution of pH 4. In an aqueous solution of pH 4, the PANI hollow spheres had a negative ζ-potential, −31.5 mV. The surface negative charge was, therefore, favorable for the deposition of positively charged PAH, and subsequent deposition of negatively charged PSS changed the ζ-potential back to a negative value (−45.0 mV); this enhanced the surface negative charge and improved the stability of the PANI hollow spheres. Deposition of Fe_3_O_4_ nanoparticles on the PE@PANI hollow spheres with an outer layer of PSS then changed the ζ-potential from −45.0 to 29.4 mV. This ζ-potential was obtained for Fe_3_O_4_ nanoparticles in aqueous solution at pH 4. Clearly, the Fe_3_O_4_ nanoparticles were easily loaded onto the PANI hollow sphere surfaces by electrostatic assembly. The specific synthesis procedures are depicted in Scheme 2. This approach has several advantages: it is controllable, general, gives high loading, and the nanoparticles are stable (Gao et al. 2006).

### Morphology and structure of Fe_3_O_4_–PE@PANI nanocomposites

**Fig. 2:**
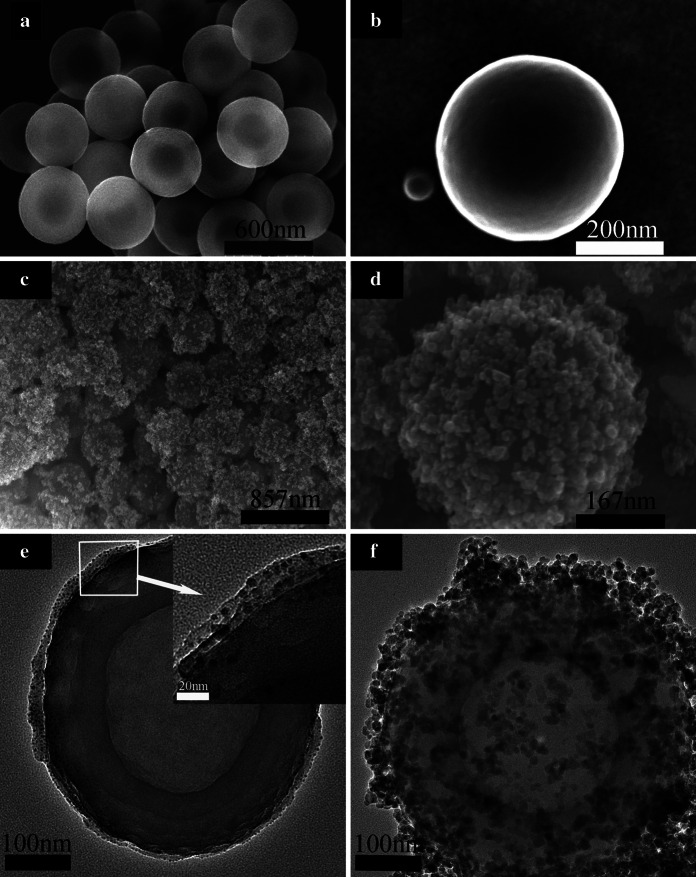
Representative FE-SEM images of PANI hollow spheres (**a** and **b**) and Fe_3_O_4_–PE@PANI nanocomposites (**c** and **d**), and TEM image of PE@PANI (**e**) and Fe_3_O_4_–PE@PANI nanocomposites (**f**)

The morphology and structure of the Fe_3_O_4_–PE@PANI nanocomposites were observed using FE-SEM and TEM. Figure 2a shows the morphology of pristine PANI hollow spheres; the surfaces of the pristine PANI hollow spheres are featureless, but they are very uniform, and the average size is about 450 nm. The PANI hollow spheres are very smooth and there are no surface impurities, as shown by the magnified FE-SEM image of a single PANI hollow sphere (Fig. 2b). Figure 2c shows the morphology of the Fe_3_O_4_–PE@PANI nanocomposites; the surfaces of the PANI hollow spheres are uniformly covered with Fe_3_O_4_ nanoparticles, and the average size of Fe_3_O_4_–PE@PANI nanocomposites is about 500 nm. Loaded nanoparticles can be clearly observed in the magnified image, and some nanoparticles are assembled into nanoclusters, but are still stably adhered to the PANI hollow sphere surfaces (Fig. 2d). After the deposition of positively charged PAH and negatively charged PSS on the PANI hollow sphere surfaces by electrostatic interactions, a core–shell structure was formed, and the TEM images show that the PANI hollow spheres were enwrapped by a layer of polymer chains of several nanometers thickness (ca. 5–20 nm; Fig. 2e). The structure of the Fe_3_O_4_–PE@PANI nanocomposites is further confirmed by TEM (Fig. 2f); the image clearly shows hollow Fe_3_O_4_–PE@PANI nanocomposites, with nanoparticles tightly and completely attached to the PANI hollow sphere surfaces.

**Fig. 3:**
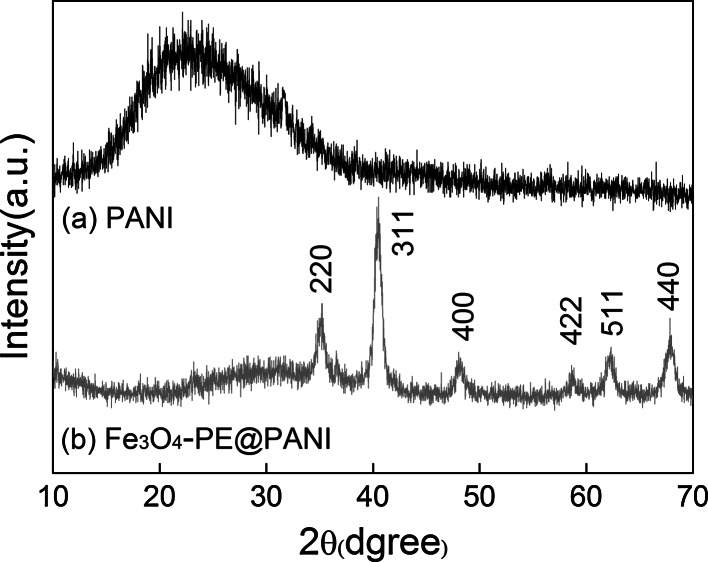
XRD patterns of PANI hollow spheres and Fe_3_O_4_–PE@PANI nanocomposites

The XRD patterns of the PANI hollow spheres and Fe_3_O_4_–PE@PANI nanocomposites are shown in Fig. 3. The PANI hollow spheres show a wide amorphous peak centered at around 2*θ* = 22°. A wide peak centered at around 22° was also observed for the Fe_3_O_4_–PE@PANI nanocomposites, together with diffraction peaks at 2*θ* = 30.1°, 35.5°, 43.3°, 53.6°, 57.2°, and 62.7°; these peaks, respectively, correspond to the (220), (311), (400), (422), (511), and (440) Bragg reflections characteristic of Fe_3_O_4_ nanoparticles. These results indicate that the Fe_3_O_4_ nanoparticles had a cubic spinel structure identical to that of the reference material (JCPDS file, PDF No. 65-3107) (Shen et al. 2010; Wei et al. 2012a, b; Bajpai and Gupta 2010). Clearly, the Fe_3_O_4_ nanoparticles were successfully coated on the surfaces of the PANI hollow spheres.

**Fig. 4:**
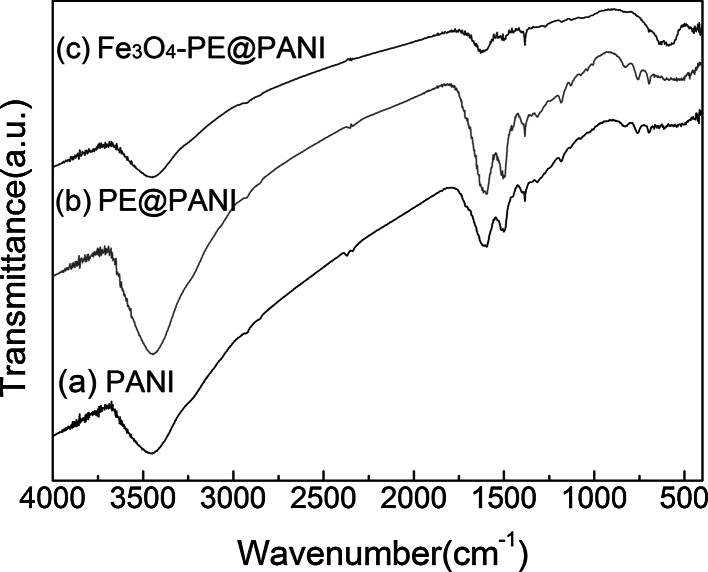
FT-IR spectra of PANI hollow spheres, PE@PANI hollow spheres, and Fe_3_O_4_–PE@PANI nanocomposites

To determine the molecular structure of the composites, we performed FT-IR analyses of the PANI hollow spheres, PE@PANI hollow spheres, and Fe_3_O_4_–PE@PANI nanocomposites. The spectra are shown in Fig. 4. Figure 4a displays the expected primary absorption features of neat PANI: the characteristic peaks at 1,594; 1,500; and 1,307 cm^−1^ can be assigned to the stretching mode of the N=quinine (Q)=N ring, N-benzene (B)–N ring, and the C–N (C_aromatic_–N) deformation, respectively (Baibarac et al. 2003; Markovic et al. 2006). The peak at 1,182 cm^−1^ results from the B–NH^+^=vibration mode (Pan et al. 2006). The absorption at around 900–700 cm^−1^ is from the aromatic ring and the out-of-plane C–H deformation vibration. The PE@PANI hollow spheres (Fig. 4b) not only show the characteristic peaks of the PANI hollow spheres, but also have a weak characteristic absorption peak at 1,228 cm^−1^, attributed to the sulfonate group (Lee et al. 2008). This observation further confirmed the presence of polyelectrolyte coated on the surfaces of the PANI hollow spheres. The spectrum of the Fe_3_O_4_–PE@PANI nanocomposites (Fig. 4c) shows a characteristic peak at 588 cm^−1^, associated with the Fe–O bond stretching, and weak peaks characteristic of PANI, confirming good coverage by Fe_3_O_4_ nanoparticles of the PANI hollow sphere surfaces.

**Fig. 5:**
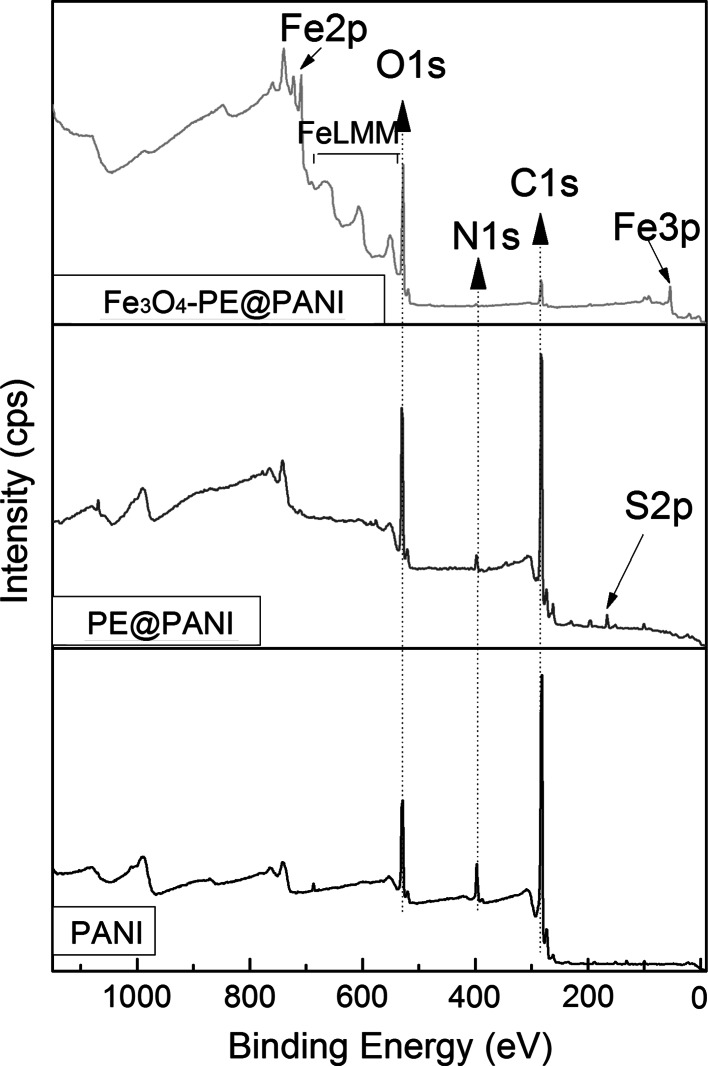
XPS wide scan of PANI hollow spheres, PE@PANI hollow spheres, and Fe_3_O_4_–PE@PANI nanocomposites

The chemical compositions of the composites were determined using XPS analysis. XPS wide-scan spectra of the PANI hollow spheres, PE@PANI hollow spheres, and Fe_3_O_4_–PE@PANI nanocomposites are shown in Fig. 5. XPS analysis revealed that the PANI hollow spheres are mainly composed of oxygen (O1s), nitrogen (N1s), and carbon (C1s). The survey scans of the PE@PANI hollow spheres not only have these elements, but also have sulfur (S2p). In addition, the relative concentration of N1s is lower in the PE@PANI hollow spheres. These results indicated deposition of polyelectrolyte on the surfaces of the PANI hollow spheres. The survey scans of the Fe_3_O_4_–PE@PANI nanocomposites revealed the presence of iron, and the relative concentrations of N1s and C1s decreased in the composites. This demonstrated that the Fe_3_O_4_ nanoparticles coated the PANI hollow sphere surfaces well. These results are in good agreement with those of the FT-IR analyses.

**Fig. 6:**
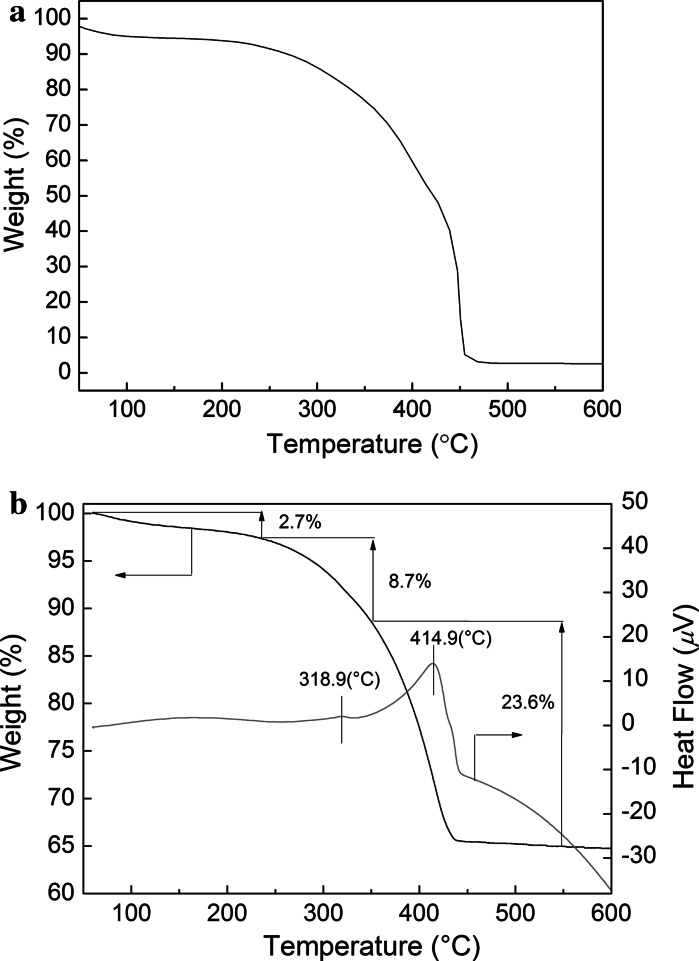
TGA of PANI hollow spheres (**a**) and Fe_3_O_4_–PE@PANI nanocomposites (**b**)

To confirm the contents of Fe_3_O_4_ nanoparticles in the resulting nanocomposites, TGA and differential thermal gravimetry were used; Thermal decomposition of PANI hollow spheres and Fe_3_O_4_–PE@PANI nanocomposites is shown in Fig. 6. As shown in Fig. 6a, it is found that the sharp weight loss of PANI hollow spheres beginning at higher than 400 °C presumably corresponded to thermal decomposition of the molecular main chains. As shown in Fig. 6b, it can be seen that three weight-loss steps take place over the scanning temperature range from 60 to 600 °C. The first weight-loss step (about 2.7 %), from 60 to 230 °C, is relatively gentle, and is related to the loss of surface-absorbed water, and the second one (about 8.7 %), between 230 and 340 °C, may arise from polyelectrolyte combustion. The third weight-loss step (about 23.6 %), from 340 to 600 °C, is a relative step, which is contributed by the PANI hollow spheres. The degradation temperatures of the polyelectrolyte and PANI hollow spheres are 318.9 °C and 414.9 °C, respectively, so about 8.7 % of polyelectrolyte is coated on the PANI hollow sphere surfaces. The Fe_3_O_4_ content, which can be calculated from the Fe_2_O_3_ mass percentage, is about 62.9 %.

### Magnetic properties of Fe_3_O_4_–PE@PANI nanocomposites

**Fig. 7:**
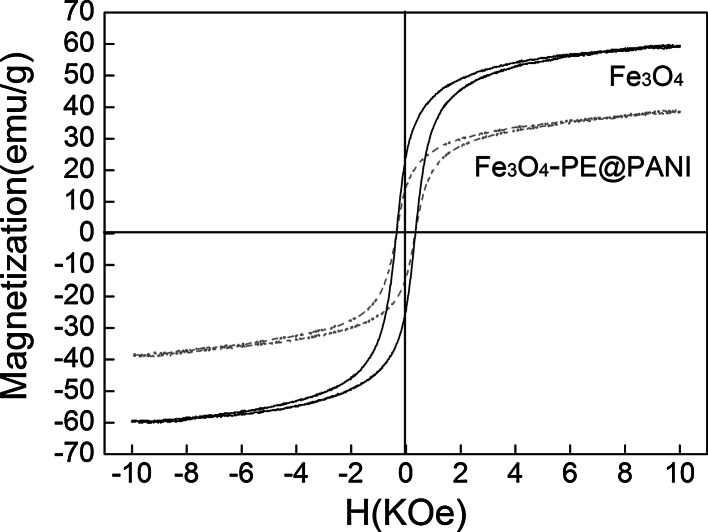
Magnetization versus applied magnetic field at room temperature for Fe_3_O_4_ nanoparticles and Fe_3_O_4_–PE@PANI nanocomposites

The magnetic properties of Fe_3_O_4_ and the Fe_3_O_4_–PE@PANI nanocomposites were measured using a vibrating sample magnetometer at room temperature, with an applied field of −10 kOe ≤ *H* ≤ 10 kOe. As shown in Fig. 7, the saturated magnetizations (*M*_s_) of pure Fe_3_O_4_ nanoparticles and the Fe_3_O_4_–PE@PANI nanocomposites are 59.6 and 38.6 emu g^−1^, respectively. The lower *M*_s_ of the Fe_3_O_4_–PE@PANI nanocomposites is caused by the presence of non-Fe_3_O_4_ polymers. Also, the magnitude of the reduction in the *M*_s_ of the Fe_3_O_4_–PE@PANI nanocomposites compared to that of pure Fe_3_O_4_ nanoparticles is basically consistent with the mass ratio (62.9 %) of Fe_3_O_4_ in the Fe_3_O_4_–PE@PANI nanocomposites. Moreover, the hysteresis curves of the Fe_3_O_4_–PE@PANI nanocomposites showed ferromagnetic behavior, with a very small coercive force (*H*_c_, about 300 Oe), so the nanocomposites are potentially useful in many applications (Zhang et al. 2009a, b).

### Electromagnetic wave absorption properties of Fe_3_O_4_–PE@PANI nanocomposites

**Fig. 8:**
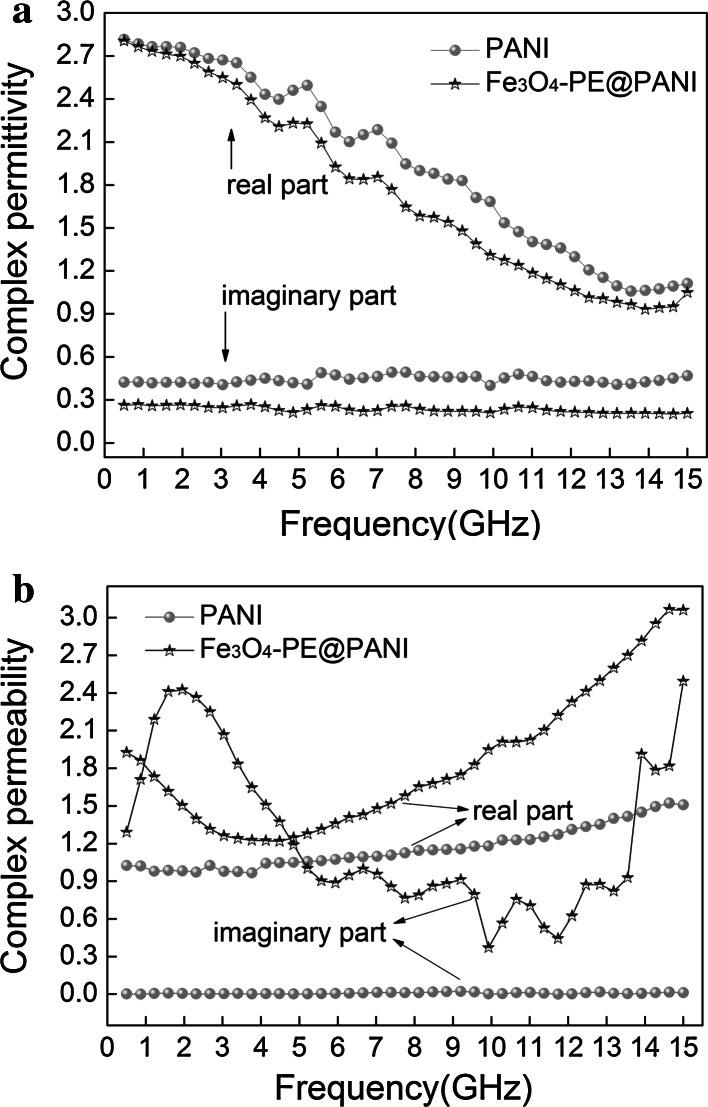
Complex permittivity (**a**) and complex permeability (**b**) of PANI hollow spheres/paraffin composite and Fe_3_O_4_–PE@PANI/paraffin composite with a weight fraction of 40 wt%

The mechanism of microwave energy loss in a material is the result of its dielectric and magnetic properties, which depends on the imaginary part of the complex permittivity and complex permeability (Kong et al. 2010). It is, therefore, necessary to investigate the complex permittivity and complex permeability. As is known, the real (*ε*′, *μ*′) and imaginary (*ε*″, *μ″*) parts of the complex permittivity and complex permeability characterize the storage ability and the loss of electric and magnetic energy, respectively, of a material (Cui et al. 2012; Li et al. 2011). The curves of the complex permittivity and complex permeability of PANI hollow spheres and Fe_3_O_4_–PE@PANI nanocomposites with a weight fraction of 40 wt% are shown in Fig. 8. It is observed that the PANI hollow spheres show higher values of *ε*′ and *ε*″ (Fig. 8a), which may be related to strong polarization as a result of the presence of polarons/bipolarons. Moreover, the *ε*′ values of the PANI hollow spheres and Fe_3_O_4_–PE@PANI nanocomposites obviously decrease with increasing frequency in the range 0.5–15 GHz. This is mainly a result of the changes in the polarizabilities and electric displacements of the materials not keeping up with the changing frequency (Wang et al. 2008). The *ε*″ values of the PANI hollow spheres and the Fe_3_O_4_–PE@PANI nanocomposites are almost constant across the whole frequency range, indicating that the *ε*″ values of the samples are less sensitive to the frequency. Figure 8b shows the real part (*μ*′) and imaginary part (*μ*″) of the samples in the frequency range 0.5–15 GHz. The PANI hollow spheres show almost constant values of the real and imaginary parts of the complex permeability across the whole frequency range, and are close to 1 and 0, respectively, indicating that magnetic loss is negligible and most absorption comes from dielectric loss. However, the real part (*μ*′) for the Fe_3_O_4_–PE@PANI nanocomposites dips slightly, and then rises with increasing frequency. The imaginary part (*μ*″) for the Fe_3_O_4_–PE@PANI nanocomposites shows a large resonance peak at a frequency of about 2 GHz, and has several small resonance peaks near 6.5, 9, 10.8, and 14 GHz. This confirms that Fe_3_O_4_–PE@PANI nanocomposites possess magnetic natural resonance in the gigahertz frequency range and magnetic loss abilities. It is expected that the derived magnetic loss together with the dielectric loss will effectively enhance the reflection loss of the electromagnetic wave.

To further reveal the microwave absorption properties of PANI hollow spheres and Fe_3_O_4_–PE@PANI nanocomposites, the reflection loss (RL) was calculated based on transmission line theory, as follows:

1 $$ {\text{RL}} = 20\log \left| {\frac{{Z_{\text{in}} - 1}}{{Z_{\text{in}} + 1}}} \right| $$

**Fig. 9:**
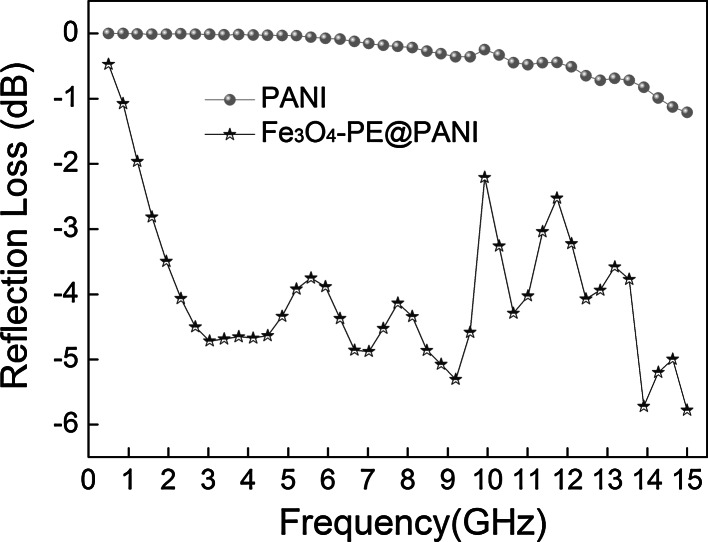
Reflection loss curves for PANI hollow spheres/paraffin composite and Fe_3_O_4_–PE@PANI/paraffin composite with a weight fraction of 40 wt% in the frequency range 0.5–15 GHz at the thickness of 2 mm

**Fig. 10:**
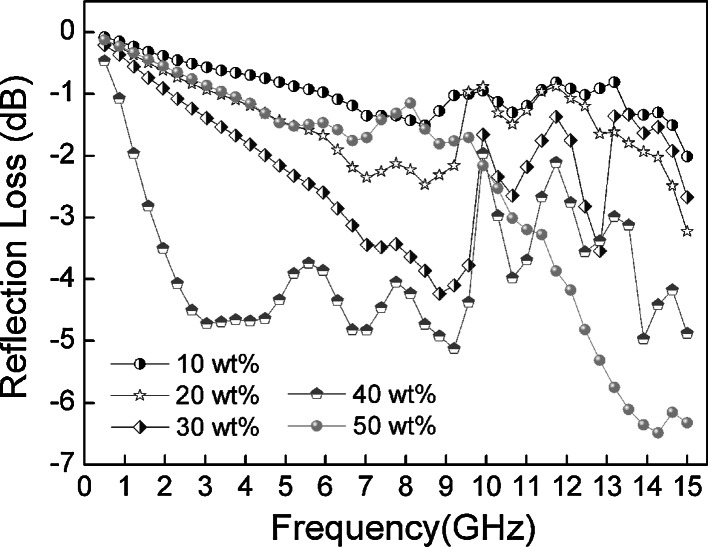
Reflection loss curves for different weight fractions of Fe_3_O_4_–PE@PANI/paraffin composite in the frequency range 0.5–15 GHz at the thickness of 2 mm

The normalized input impedance (*Z*_in_) is given by the formula

2 $$ Z_{\text{in}} = \sqrt {\frac{{\mu_{\text{r}} }}{{\varepsilon_{\text{r}} }}} \tanh \left[ {j\left( {\frac{2\pi fd}{c}} \right)\sqrt {\mu_{\text{r}} \varepsilon_{\text{r}} } } \right] $$

where *ε*_r_ = *ε*′ − *jε″*, *μ*_r_ = *μ*′ − *jμ*″, *f* is the microwave frequency in hertz, *d* is the thickness of the absorber in m, and *c* is the velocity of light in free space in m/s. The reflection loss of different weight fractions of Fe_3_O_4_–PE@PANI/paraffin composites was measured at a sample thickness of 2 mm. Herein, the reflection loss of Fe_3_O_4_–PE@PANI nanocomposites is compared with PANI hollow spheres with a weight fraction of 40 wt%; the results are shown in Fig. 9. It can be observed that the Fe_3_O_4_–PE@PANI nanocomposites exhibit distinguishable reflection loss abilities and a wide response bandwidth. The minimum reflection loss of the Fe_3_O_4_–PE@PANI nanocomposites is more than −5 dB, near 4, 9, and 14–15 GHz. It can also be seen that the Fe_3_O_4_–PE@PANI nanocomposites maintained excellent reflection loss (approximately −5 dB) in the range 2.5–15 GHz, compared with that of PANI hollow spheres. It is noteworthy that the PANI hollow spheres only display a minimum reflection loss at about −1.2 dB at 15 GHz, although they have larger *ε*′ and *ε*″ values than the Fe_3_O_4_–PE@PANI nanocomposites. Figure 10 shows the reflection loss of different weight fractions of Fe_3_O_4_–PE@PANI/paraffin composite with a thickness of 2 mm in the frequency range 0.5–15 GHz. It can be seen that the reflection loss properties are sensitive to the content of Fe_3_O_4_–PE@PANI in the composites, and the reflection loss properties toward incident electromagnetic wave of samples are enhanced substantially with increase in the content of Fe_3_O_4_–PE@PANI. Meanwhile, the frequency relating to the minimum reflection loss can also be modulated by the content of Fe_3_O_4_–PE@PANI in the composites. The Fe_3_O_4_–PE@PANI/paraffin composite shows a minimum reflection loss of −6.5 dB at 14.3 GHz for a weight fraction of 50 wt%, and the frequency bandwidth at less than −5 dB is from 12.5 to 15 GHz. Enhancement in microwave absorption of Fe_3_O_4_–PE@PANI/paraffin composite with increase in the content of Fe_3_O_4_–PE@PANI can be explained as follows. Increasing of Fe_3_O_4_–PE@PANI nanocomposites content effectively increases the complex permittivity/complex permeability, leading to matched characteristic impedances and dielectric/magnetic loss abilities. Moreover, the consequent nanocomposites interfaces will produce interfacial relaxation between Fe_3_O_4_ nanoparticles and PANI hollow spheres, which is also beneficial to microwave absorption. As mentioned above, Fe_3_O_4_–PE@PANI nanocomposites show excellent reflection loss, are lightweight, and have large absorbing bandwidths, and are, therefore, promising for a variety of technological applications.

## Conclusion

A facile approach to the synthesis of Fe_3_O_4_–PE@PANI nanocomposites was presented. A polyelectrolyte film was successfully coated onto PANI hollow sphere surfaces. Based on the strong electrostatic attractions between opposite charges and other interactions, paramagnetic Fe_3_O_4_ nanoparticles were assembled on the convex surfaces of PE@PANI, affording paramagnetic nanocomposites. This approach has the advantages of generality, reproducibility, controllability, tailorability, high loading capability, and stability, and is promising for producing a wide range of functional nanocomposites. The Fe_3_O_4_–PE@PANI nanocomposites exhibited excellent magnetic properties and produced magnetic resonance and loss in the nanocomposites. The reflection loss, calculated using the absorbing-wall theory, showed that the Fe_3_O_4_–PE@PANI nanocomposites exhibited better reflection loss abilities and wider response bandwidths than those of PANI hollow spheres in the range 0.5–15 GHz. In summary, the synthesized electromagnetically functionalized Fe_3_O_4_–PE@PANI nanocomposites are promising for applications in microwave-absorbing materials.
